# The research of a novel WOG-YOLO algorithm for autonomous driving object detection

**DOI:** 10.1038/s41598-023-30409-1

**Published:** 2023-03-06

**Authors:** Lingzhi Xu, Wei Yan, Jiashu Ji

**Affiliations:** School of Energy and Power Engineering, Shandong University, Jinan, 250000 China

**Keywords:** Electrical and electronic engineering, Computer science

## Abstract

Object detection has been one of the critical technologies in autonomous driving. To improve the detection precision, a novel optimization algorithm is presented to enhance the performance of the YOLOv5 model. First, by improving the hunting behavior of the grey wolf algorithm(GWO) and incorporating it into the whale optimization algorithm(WOA), a modified whale optimization algorithm(MWOA) is proposed. The MWOA leverages the population’s concentration ratio to calculate $p_h$ for selecting the hunting branch of GWO or WOA. Tested by six benchmark functions, MWOA is proven to possess better global search ability and stability. Second, the C3 module in YOLOv5 is substituted by G-C3, and an extra detection head is added, thus a highly optimizable detection G-YOLO network is constructed. Based on the self-built dataset, 12 initial hyperparameters in the G-YOLO model are optimized by MWOA using a score fitness function of compound indicators, thus the final hyperparameters are optimized and the whale optimization G-YOLO (WOG-YOLO) model is obtained. In comparison with the YOLOv5s model, the overall mAP increases by 1.7$\%$, the mAP of pedestrians increases by 2.6$\%$ and the mAP of cyclists increases by 2.3$\%$.

## Introduction

Autonomous driving integrates environmental perception, dynamic planning, and control execution in automobiles, it has received considerable scholarly attention in recent years^1^. Object detection serves as the principal perception method for autonomous vehicles, and the crux of this task is to enhance the accuracy of object detection.

Object detection algorithms can be divided into two major classes: two-stage detectors e.g. Faster R-CNN^2^, TS4Net^3^, AccLoc^4^, and Part-$A^ 2$ net^5^, and one-stage detectors e.g. YOLO^6^, CG-SSD^7^, and PAOD^8^. Two-stage algorithms generate region proposals, then classify and localize objects according to them. Contrary to two-stage algorithms, one-stage ones perform classification and localization using pre-defined candidate proposals. In general, two-stage detectors achieve greater accuracy but are more time-consuming than one-stage detectors.

To obtain a more robust and accurate detection model, the following literature provides different improvement methods. Shi et al.^9^ introduced GIoU into K-means++ to obtain better anchors. Manuel et al.^10^ used an evolutionary algorithm to search for optimal region-based anchors. Wang et al.^11^ proposed a feature extraction network to ensure that small objects are correctly detected. Wang et al.^12^ adopted the dynamic attention module to improve detection performance. As a model with arbitrary hyperparameters leads to unsatisfactory performance, optimization algorithms e.g. Bayesian optimization^13^, and fitness sorted rider optimization algorithm^14^ have been used to find the optimal hyperparameter group.

How to quickly and accurately obtain the optimal high-dimensional parameter combination optimization is a major problem, the metaheuristic optimization algorithms such as ant colony optimization(ACO)^15^, particle swarm optimization(PSO)^16^, whale optimization algorithm(WOA)^17^, grey wolf optimization(GWO)^18^, and firefly algorithm(FA)^19^ aim to solve this. WOA is known for its simplicity and outstanding global solving ability among a variety of optimization algorithms, it has been applied in solving the optimal hyperparameter group^20–22^, data clustering^23–26^, multi-objective problems^27,28^, etc. However, the performance of canonical WOA is limited by low convergence and unsatisfactory accuracy. Therefore, WOA must be improved by weighing up exploration and exploitation^29,30^, integrating other algorithms^31–33^, and using better update strategies^34,35^. For example, in SHADE-WOA, Chakraborty et al.^36^ added an extra parameter $\alpha$ which is used to control the exploration and exploitation phases. In WhaleFOA^37^, the original FOA’s random search strategy is replaced by WOA’s hunting strategy to enrich FOA’s global exploration capability. Chen et al.^38^ developed a double adaptive weight strategy, the results show that the WOA using this method has better global optimization capability.

Referring to the above literature, a novel modified whale optimization algorithm(MWOA) is proposed by fusing the structure of WOA and the hunting strategy of GWO with multi-faceted improvements. The core procedures of MWOA are as follows: the scaling factor is calculated using an adaptive update formula based on the population’s fitness. To improve GWO’s optimization performance, the position of the local optima is added as one instructor along with $\alpha$ wolf, $\beta$ wolf, and $\delta$ wolf in the GWO’s hunting strategy. The instruction vectors $V^t_{k,i}$ are improved using the new formula and they are weighted by fitness. Then the population’s concentration ratio is leveraged as the controller of the MWOA’s hunting branches. To verify the performance of MWOA, six multi-dimensional benchmark functions are used as the fitness function. The test result shows that MWOA possesses better performance. A novel G-YOLO network is proposed and MWOA is implemented to optimize its hyperparameters. A self-built dataset including pedestrians, cyclists, and cars serves as the training set and test set of G-YOLO, and the final whale optimization G-YOLO(WOG-YOLO) model presents a stronger detection ability and stability.

## Proposed MWOA algorithm

### Description of WOA and GWO

The canonical WOA is enlightened by the foraging mechanism of the humpback whales, it defines three behaviors to search for the best global solution. Its optimization stages can be concluded as follows: initialize the population and related parameters, calculate each individual’s fitness and identify the best global solution, then update the individuals’ position through the following formulas:

1 $$\begin{aligned} \vec {X}_i^{t+1}= & {} \left\{ \begin{array}{lll} V_i^t &{}&{} {p_h<0.5,|A|<1}\\ V_{rand}^t &{}&{} {p_h<0.5,|A|\ge 1}\\ \vec {X^t_*}+|\vec {X^t_*}-\vec {X^t_i}|\cdot e^{bl}\cdot cos(2\pi l) &{} &{} {p_h\ge 0.5}\\ \end{array} \right. \end{aligned}$$

2 $$\begin{aligned} \vec {V_i^t}= & {} \vec {X_*^t}-A\cdot |C\cdot \vec {X_*^t}-\vec {X_i^t}| \end{aligned}$$

3 $$\begin{aligned} A= & {} 2a\cdot r_{1}-a \end{aligned}$$

4 $$\begin{aligned} C= & {} 2\cdot r_{2} \end{aligned}$$

5 $$\begin{aligned} a= & {} 2-\frac{2t}{T} \end{aligned}$$

where $\vec {X_i^t}$ and $\vec {X}_i^{t+1}$ are the positions of *i* th individual in *t* th and $t+1$ th iteration respectively, $\vec {X^t_*}$ is the optimal position of in *t* th iteration, $p_h$, $r_1$, $r_2$ are random numbers in the range of [0, 1], *l* is a random number in the range of $[-1,1]$, *b* is the spiral constant(in this paper, *b* equals 1), and *a* is the scaling factor that hinges on the current iteration step *t* and the maximum number of iteration steps *T*.

Repeat the above steps until the end requirements are satisfied.

The standard GWO selects $\alpha$ wolf, $\beta$ wolf, and $\delta$ wolf from the wolf pack by each individual’s fitness, then updates the individual’s position by the following formulas:

6 $$\begin{aligned} \vec {X}^{t+1}_i= & {} \frac{\vec {V_{\alpha ,i}^t}+\vec {V_{\beta ,i}^t}+\vec {V_{\delta ,i}^t}}{3} \end{aligned}$$

7 $$\begin{aligned} \vec {V_{k,i}^t}= & {} \vec {X_k^t}-A\cdot |C\cdot \vec {X_k^t}-\vec {X_i^t}| \end{aligned}$$

where $\vec {X_i^t}$ and $\vec {X}_i^{t+1}$ are the positions of *i* th individual in *t* th and $t+1$ th iteration respectively, $\vec {X_k^t}$ is the position of *k*, *k* can represent the position of $\alpha$ wolf, $\beta$ wolf, and $\delta$ wolf, *A* and *C* are the same as the formula in WOA.

### Modified WOA

#### Adaptive scaling factor

In WOA and GWO, the scaling factor decreases linearly to control the process of conversion from global optimation to local optimation. However, this approach fails to accommodate the practical condition as most optimization problems are complicated non-linear processes. Hence an adaptive scaling factor formula is proposed as follows:

8 $$\begin{aligned} a=2-min\{2,\frac{f^{t-1}_*}{f^t_*}(1-cos\frac{t\pi }{T})\} \end{aligned}$$

where $f^t_*$ and $f^{t-1}_*$ are the optimal fitness in the current iteration and last iteration respectively.

In the above formula, the scaling factor is modulated by $\frac{f^{t-1}_*}{f^t_*}$, thus expanding the searching scope if $\frac{f^{t-1}_*}{f^t_*}<1$ or vice versa if $\frac{f^{t-1}_*}{f^t_*}\ge 1$. The cosine function introduces non-linearity into the scaling factor. Furthermore, the minimum value function is used to ensure the scaling factor is greater than or equals to zero.

#### Improved GWO’s hunting strategy

In WOA, the optimal individual’s position instructs the update of other individuals’ positions. This method can facilitate convergence but has poor robustness, namely, it may stagnate around the local optimal solution. To accelerate the convergence of local optimation and strengthen the ability to search for global solutions, the position update formula of GWO is introduced to replace the original optimal position update method. Furthermore, each individual’s historical optimal position $\vec {X^t_l}$ is introduced to calculate $\vec {V^t_l}$. The new position update formula is as follows:

9 $$\begin{aligned} \vec {X}^{t+1}_i=w_\alpha ^t\vec {V_\alpha ^t} +w_\beta ^t\vec {V_\beta ^t}+w_\delta ^t\vec {V_\delta ^t}+w_l^t\vec {V_l^t} \end{aligned}$$

where $w_\alpha$, $w_\beta$, $w_\delta$, and $w_l$ denote the weights of $\alpha$, $\beta$, $\delta$, and optimal local position, $\vec {V_\alpha ^t}$, $\vec {V_\beta ^t}$, $\vec {V_\delta ^t}$, and $\vec {V_l^t}$ denote the instruct vectors of $\alpha$, $\beta$, $\delta$, and each individual’s historical optimal position.

The weights $w_\alpha ^t$, $w_\beta ^t$, $w_\delta ^t$, and $w_l^t$ depend on their respective positions’ fitness. Taking the case of minimal optimization, the position gets greater weight with smaller fitness, and then the weights are normalized into the range of [0, 1]. The weight can be calculated by:

10 $$\begin{aligned} w_k^t=\frac{1}{f_k^t\cdot (\sum {\frac{1}{f_j^t}})} \end{aligned}$$

where $f_k^t$ denotes the fitness in position *k*, *j* in $\sum {\frac{1}{f_j}}$ can represent the fitness of $\alpha$, $\beta$, $\delta$, or each individual’s historical optimal position *l*.

In GWO, a random number $C_i$ in the range of [0, 2] is used to control the influence of optimal position. However, this method is uncontrollable, namely that it lacks the precise trade-off between global optimization and local optimation.

To solve this issue, $|1-a|$ and $1-|1-a|$ are introduced to improve the original $\vec {V_{k,i}^t}$. $|1-a|$ is used to control the influence of $\alpha$, $\beta$, and $\delta$, $1-|1-a|$ is used to control the influence of historical optimal position *l*. As *a* decreases from 2 to 0, $|1-a|$ firstly decreases from 1 to 0, and then increases from 0 to 1, this method fully utilizes the position of $\alpha$, $\beta$, and $\delta$ and can facilitate convergence in both early and final stages. In the middle stage, $|1-a|$ is close to zero and $1-|1-a|$ is close to 1, the position update is mainly instructed by *l* and random variations, thus the population has better global optimization ability. $\vec {V_t}$ can be expressed as follows:

11 $$\begin{aligned} \vec {V_{k,i}^t}= & {} |1-a|\vec {X_{k}^t}-A*||1-a|\vec {X^t_{k}}-\vec {X^t_i}| \end{aligned}$$

12 $$\begin{aligned} \vec {V_{l,i}^t}= & {} (1-|1-a|)\vec {X_l^{t}}-A*|(1-|1-a|)\vec {X^t_l}-\vec {X^t_i}| \end{aligned}$$

where *k* in $X_{k}^t$ can be represented by $\alpha$, $\beta$, and $\delta$.

During the optimation process, the warmup skill is used: in the first $N_{warmup}$ (e.g. 2) iterations, the scaling factor is set to be very small(e.g. 0.1), and after the warmup iterations the scaling factor reverts to normal behavior. This method helps the entire population find its better optimization direction and recognize the most efficient way to enhance their fitness.

### Incorporation of improved GWO’s hunting strategy into WOA

In WOA, spiral hunting and the normal optimal position update method are used, and they both have a 50$\%$ possibility of being executed. As the spiral hunting method has a larger search scope, and the optimal position update method searches comparatively in the local scope, a new possibility $p_{h}$ is proposed. $p_{h}$ is used to serve as the possibility of the spiral hunting method, and it decreases to 0 gradually as *a* decreases. This branch control method keeps the population to be neither too concentrated nor too sparse.

13 $$\begin{aligned} p_{h}= & {} \theta ^t\cdot \frac{a}{2} \end{aligned}$$

14 $$\begin{aligned} \theta ^t= & {} \frac{N\cdot f_*^{t-1}}{\sum _{i=1}^N f_i^{t-1}} \end{aligned}$$

where *a* is mentioned above in Eq. (8), $\theta$ is the population’s concentration ratio, $\sum _{i=1}^N f_i^{t-1}$ stands for the sum of all individuals’ fitness, *N* denotes the total number in the population, and $f_*^{t-1}$ is the best fitness of the current population.

The crucial problem of swarm intelligence is that the population’s concentration ratio $\theta$ graduates to being huge. As $\theta$ gets larger, it results in narrower diversity of the population, hence making it harder to continue global optimation. Therefore, the population’s concentration ratio is calculated and leveraged to control the ratio $\theta$.

**Figure 1:**
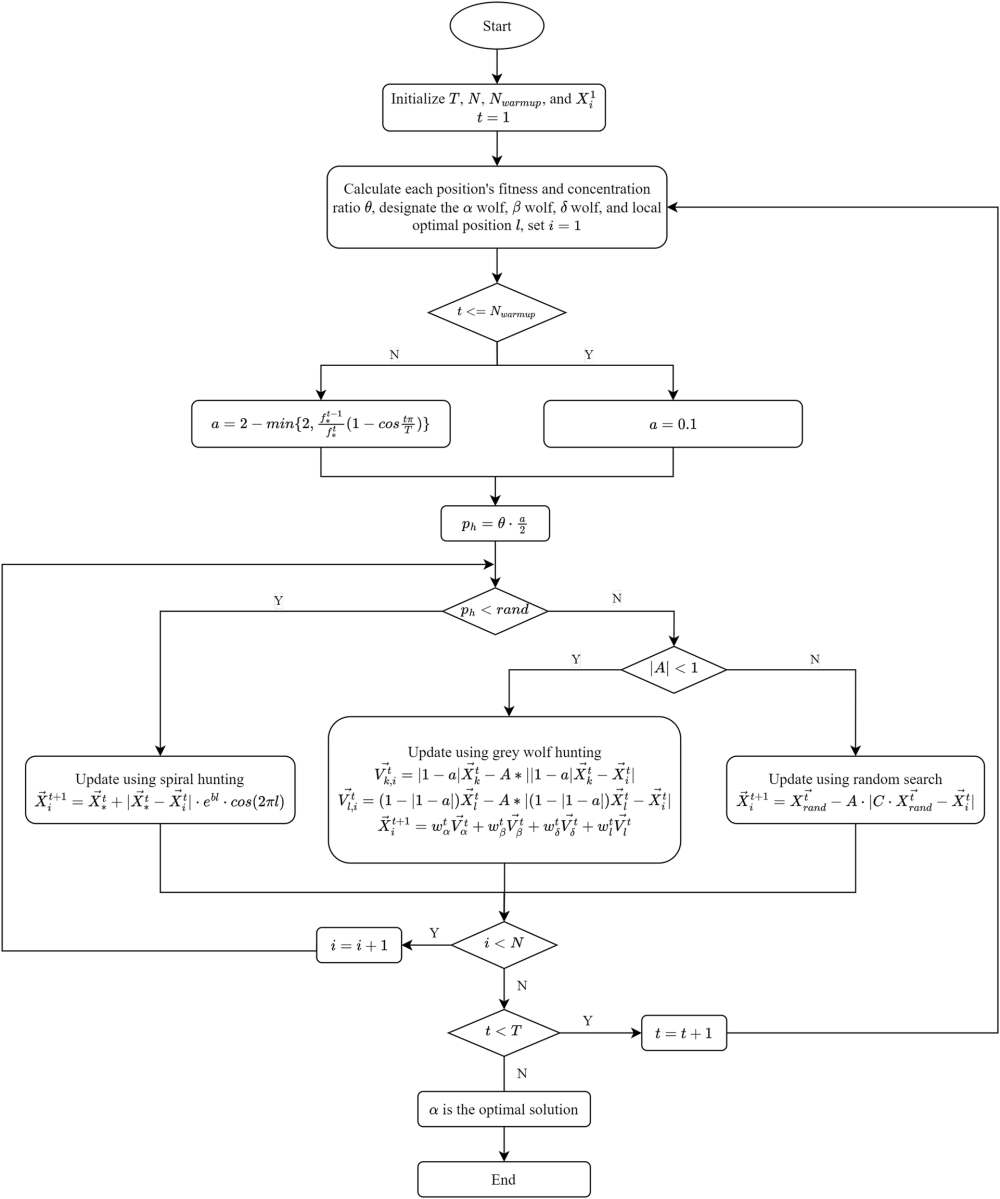
The flow diagram of MWOA.

The graphic process of MWOA is shown in the Fig. 1.

### Benchmark function test

Six multi-dimensional benchmark functions^36^ are used to verify the effectiveness and precision. F1, F2, F5, and F6 have many local minima, hence the optimization algorithm is prone to stagnate around them. F6 possesses many global mininum positions with the same value and its minimum value is determined by the dimension. F3 and F4 are bowl-shaped and they don’t have a local minimum. The hyperparameters of the target detection model are generally less than 20, thus the dimension of the test function *d* is set to 20. They are listed as follows:

15 $$\begin{aligned} F_1(x)= & {} -d e^{-0.2\sqrt{\frac{1}{d}\sum _{i=1}^{d}{x_i^2}}}-e^{\frac{1}{d}\sum _{i=1}^{d}{cos(2\pi x_i)}}+d+e \end{aligned}$$

16 $$\begin{aligned} F_2(x)= & {} \sum ^{d}_{i=1}\frac{x_i^2}{4000}-\prod ^{d}_{i=1}{cos(\frac{x_i}{\sqrt{i}})}+1 \end{aligned}$$

17 $$\begin{aligned} F_3(x)= & {} \sum ^{d}_{i=1}x_i^2+(\sum _{i=1}^{d}0.5ix_i)^2+(\sum _{i=1}^{d}0.5ix_i)^4 \end{aligned}$$

18 $$\begin{aligned} F_4(x)= & {} \sum ^{d}_{i=1}|x_i|^{i+2} \end{aligned}$$

19 $$\begin{aligned} F_5(x)= & {} \frac{1}{2}\sum ^{d}_{i=1}(x_i^4-16x_i^2+5x_i) \end{aligned}$$

20 $$\begin{aligned} F_6(x)= & {} \prod _{i=1}^{d}\left( \sum _{j=1}^{6}j\cos {(j+1)x_i+j}\right) \end{aligned}$$

PSO^16^, WOA^17^, GWO^18^, WhaleFOA^37^, and MWOA are employed to solve the above functions. In order to obtain the objective results, the common parameters are set to be consistent: the maximum number of iterations is 100, and the number of individuals in the population is 50. In PSO, the local coefficient and global coefficient are set to be 2.05, the minimum weight of the bird is 0.4, and the maximum weight of the bird is 0.8. In WhaleFOA, the safety threshold value is 0.8, and the number of producers percentage is 0.2. Test functions and MWOA are implemented using NumPy^39^. The core algorithms of PSO, GWO, WOA, and WhaleFOA are implemented by mealpy^40^.

**Figure 2:**
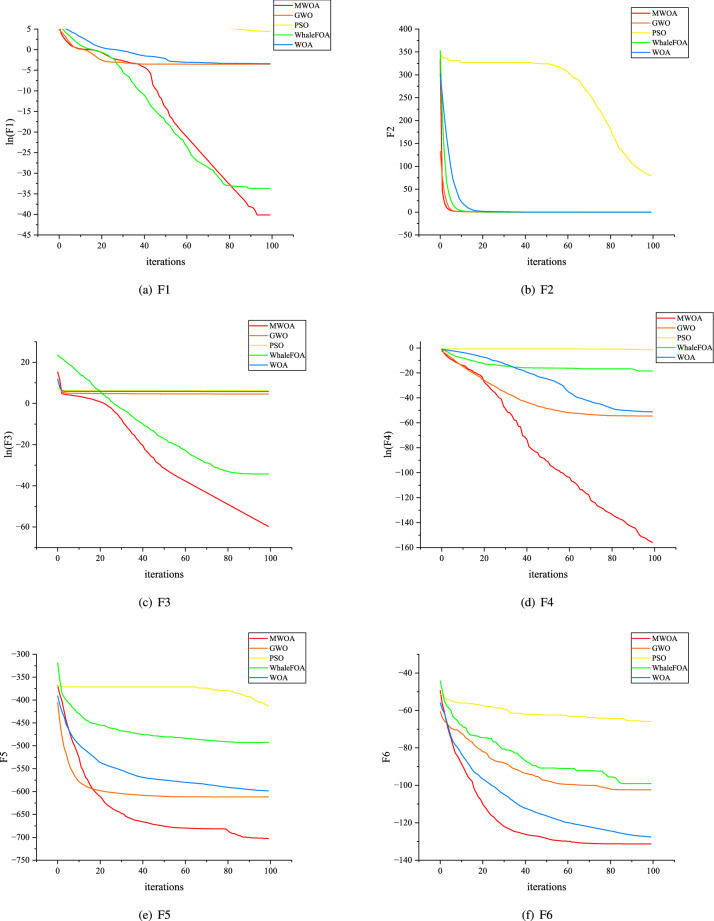
The average iteration process.

All algorithms are tested using a device with an i5-10600KF processor and 32.0 GB RAM, each benchmark function is run independently thirty times, Table 1 shows the best and worst results in thirty solutions. Dealing with F1, F2, F5, and F6, MWOA is blessed with a more robust global searching ability and hardly falls into stagnation. As for bowl-shaped problems like F3 and F4, MWOA achieves better accuracy and stability. To get a scrutiny of the iteration process, Fig. 2 provides the average convergence curves of PSO, GWO, WOA, WhaleFOA, and MWOA. Contrasting with other algorithms, MWOA possesses faster convergence faculty during both the early and final stages.

**Table 1:** Benchmark function test.

Function	Optimal solution	Algorithm	Best	Worst	Mean	Std
F1	0	MWOA	4.00E−15	4.00E−15	4.00E−15	0.00E+00
		GWO	7.96E−10	1.02E−07	1.33E−08	1.94E−08
		PSO	2.45E+00	9.10E+00	5.72E+00	1.56E+00
		WhaleFOA	2.18E−14	2.70E−10	3.17E−11	5.44E−11
		WOA	3.93E−11	1.44E−06	4.96E−08	2.58E−07
F2	0	MWOA	0.00E+00	0.00E+00	0.00E+00	0.00E+00
		GWO		8.56E−01	2.97E−02	1.54E−01
		PSO	9.06E+00	2.23E+02	7.96E+01	5.87E+01
		WhaleFOA	0.00E+00	6.87E−14	2.37E−15	1.23E−14
		WOA	0.00E+00	6.54E−01	3.33E−02	1.25E−01
F3	0	MWOA	2.44E−31	3.10E−25	1.16E−26	5.55E−26
		GWO	2.95E+01	2.22E+02	1.00E+02	4.67E+01
		PSO	1.69E+02	8.27E+02	5.13E+02	1.80E+02
		WhaleFOA	8.55E−24	2.95E−14	1.33E−15	5.28E−15
		WOA	1.89E+02	5.03E+02	3.24E+02	7.53E+01
F4	0	MWOA	1.88E−87	3.51E−67	1.85E−68	6.70E−68
		GWO	2.42E−31	4.63E−23	2.04E−24	8.36E−24
		PSO	3.16E−04	7.04E−01	2.10E−01	2.26E−01
		WhaleFOA	2.24E−18	1.55E−07	9.15E−09	3.00E−08
		WOA	1.77E−34	1.60E−21	5.34E−23	2.88E−22
F5	−39.17*d*	MWOA	−7.83E+02	−5.02E+02	−7.03E+02	7.77E+01
		GWO	−7.30E+02	−4.81E+02	−6.12E+02	5.62E+01
		PSO	−5.58E+02	−3.27E+02	−4.12E+02	5.64E+01
		WhaleFOA	−5.00E+02	−4.54E+02	−4.93E+02	9.82E+00
		WOA	−7.16E+02	−4.65E+02	−5.98E+02	5.96E+01
F6	−29.67 ($d=2$)	MWOA	−1.87E+02	−6.17E+01	−1.31E+02	2.56E+01
		GWO	−1.36E+02	−7.75E+01	−1.02E+02	1.81E+01
		PSO	−8.78E+01	−4.76E+01	−6.59E+01	8.36E+00
		WhaleFOA	−1.64E+02	−6.70E+01	−9.90E+01	2.41E+01
		WOA	−1.83E+02	−9.45E+01	−1.28E+02	1.94E+01

## WOG-YOLO

### Network structure

**Figure 3:**
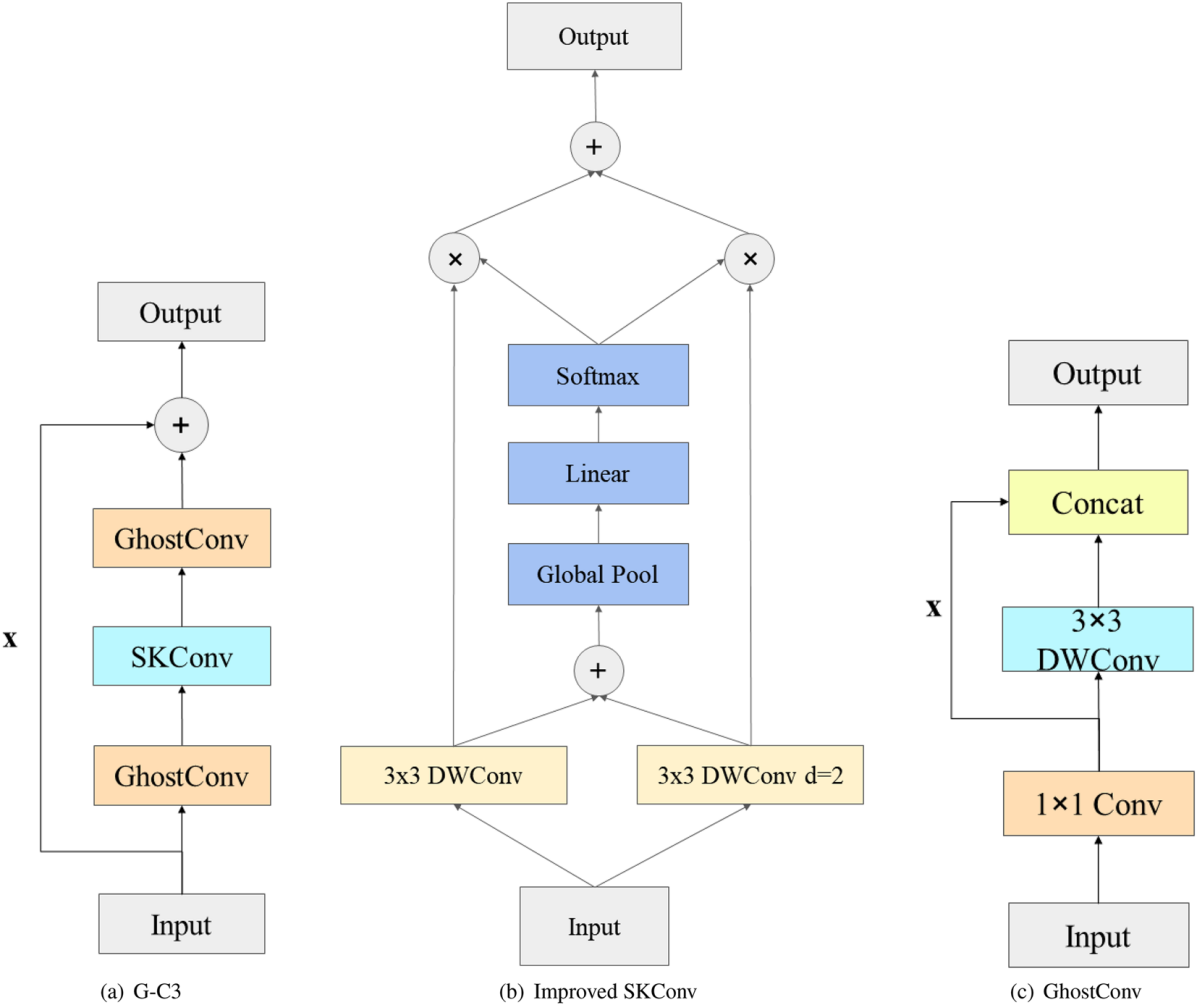
The structures of G-C3, improved SKConv and GhostConv.

YOLOv5^41^ is one of the most famous and utilitarian object detectors, it’s known for its high detection speed and elegant structure. Nevertheless, limited by its grid-based mechanism, YOLOv5 lacks competence in detecting small objects, thus the new YOLOv5 structure, named as G-YOLO, is proposed. The attention mechanism in SKNet^42^ is introduced into G-YOLO’s backbone network and the original C3 block is replaced by the G-C3 block. As convolution with a $3\times 3$ kernel is sensitive to small features and convolution with a $5\times 5$ kernel is sensitive to larger features, the SKConv can switch to the $3\times 3$ or $5\times 5$ perceptive field easily to obtain smaller scale features or bigger features. However, using the above two convolutions isn’t cost-effective compared with a single $3\times 3$ convolution. Hence depth-wise convolution^43^ is used to replace the vanilla convolution, furthermore, the $5\times 5$ convolution is replaced by $3\times 3$ convolution whose dilation is set to 2. The improved SKConv has the same perceptive field and it has fewer parameters. The structures of G-C3, improved SKConv and GhostConv are shown in Fig. 3a–c.

**Figure 4:**
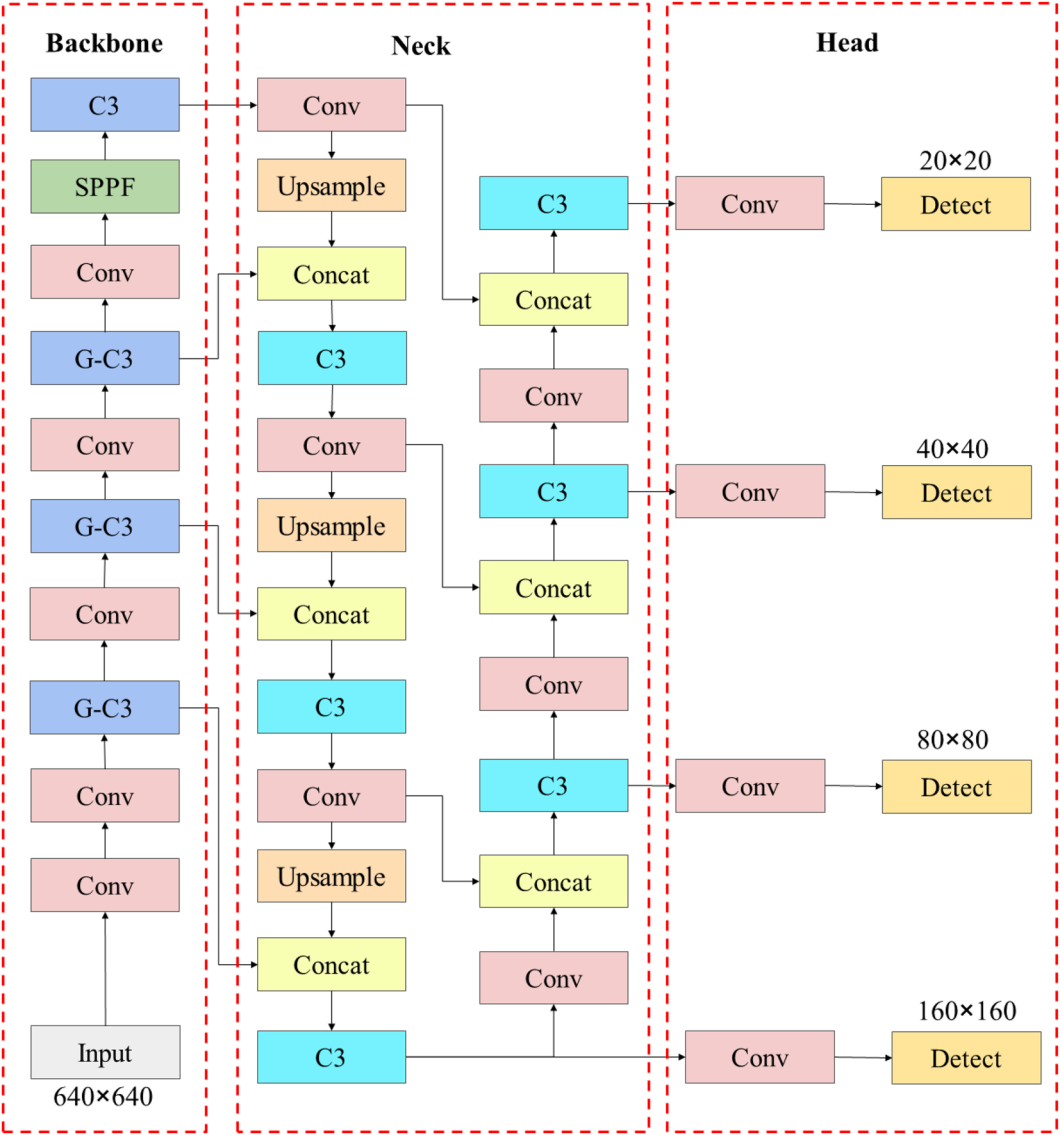
G-YOLO.

An extra detection head is added to the G-YOLO to detect small objects more efficaciously and precisely. The number of branches in the PAN structure changes from 3 to 4, namely, a new branch using the $160\times 160$ feature map is added for minor objects. The new network requires more parameters, to keep the network lightweight, GhostConv^43^ is introduced into the G-C3 block. GhostConv takes advantage of both vanilla convolution and depth-wise convolution, thus the number of trainable parameters reduces sharply without losing too much detection precision. Compared to the mostly used lightweight model YOLOv5s, the parameter size of G-YOLO is close to YOLOv5s. The new structure is shown in Fig. 4.

### Data preparation and processing

#### Dataset

A great deal of previous research into autonomous driving has focused on the detection of cars, cyclists, and pedestrians using the Kitti dataset^44^, nevertheless, the precision in pedestrians and cyclists is unsatisfactory in comparison with that of cars. The low detection accuracy is far from the practical application of autonomous driving. The images containing vehicles, cyclists and pedestrians are extracted from the Kitti dataset, then vans and trucks are labelled cars. The final dataset contains 5325 images.

#### Data augmentation

In YOLOv5, mosaic is used as an image augment method, which gives the YOLOv5 network considerable enhancement in both precision and recall. The input image should be square in YOLOv5, but the width and height of images in the Kitti dataset are 1240 and 370 pixels respectively. Thus a large part of the input image is padded with blank. To reduce the padded area, three images are concatenated vertically as one image before using the mosaic method.

### Optimization of YOLOv5’s hyperparameter

Parameters function as a critical part of the convolution network, it controls the entire training process and has a great impact on the performance of the final detection model. For models with a brand-new framework, tuning parameters one by one can be time-consuming and inefficient. Furthermore, inadequately tuned parameters can not fully reflect the performance of the model. Fine-tuned parameters can boost the recall and precision by setting a suitable threshold which is instrumental in obtaining a performant model.

In this paper, 12 parameters in G-YOLO are chosen to evolve using MWOA, their names and ranges are shown in Table 2. To be cost-efficient, the number of individuals is set to 5, the iteration number is set to 10.

**Table 2:** 12 hyperparameters in YOLOv5.

Hyperparameter	Description	Lower limit	Uppper limit
lr0	Initial learning rate	1.00E−05	1.00E−01
lrf	Final OneCycleLR learning rate	0.01	1
Momentum	SGD momentum	0.6	0.98
Weight_decay	Optimizer weight decay	0	0.001
Warmup_epochs	Warmup epochs	0	5
Warmup_momentum	Warmup initial momentum	0	0.95
Warmup_bias_lr	Warmup initial bias lr	0	0.2
Box	Box loss gain	0.02	0.2
Cls	Class loss gain	0.2	4
Cls_pw	Class BCELoss positive_weight	0.5	2
Obj	Object loss gain	0.2	4
Obj_pw	Object BCELoss positive_weight	0.5	2

The fitness function receives the newcome parameters, dispatches them to G-YOLO, and then activates the training of the detection model. After the G-YOLO training process, the evaluation score of the model is passed back to MWOA as the fitness. Trained with the optimal hyperparameters, the final whale optimization G-YOLO (WOG-YOLO) model is obtained.

The evaluating indicators are *P*, *R*, *F*1, and *mAP*. *P* refers to precision, which calculates the ratio of the number of correct detection results *TP* and the number of total detection results($TP+FP$). *R* refers to recall, which calculates the ratio of the number of correct detection results *TP* and the number of actual objects($TP+FN$). *F*1 is based on the harmonic mean of *P* and *R*, which considers both *P* and *R*. The indicators and scores are calculated by following formulas:

21 $$\begin{aligned} P= & {} \frac{TP}{TP+FP} \end{aligned}$$

22 $$\begin{aligned} R= & {} \frac{TP}{TP+FN} \end{aligned}$$

23 $$\begin{aligned} F1= & {} 2\cdot \frac{P\times R}{P+R} \end{aligned}$$

24 $$\begin{aligned} AP= & {} \int _0^1P(R)dR \end{aligned}$$

25 $$\begin{aligned} mAP= & {} \frac{\sum _{i=1}^{k}AP_i}{k} \end{aligned}$$

26 $$\begin{aligned} score= & {} \frac{1}{0.1P+0.1R+0.2F1+0.6mAP} \end{aligned}$$

where k is the number of classes.

## Results

The experiment is based on Ubuntu 18.04, using NVIDIA A2000 GPU. The batch size is 8, the number of training epochs is 100, the image size is $640\times 640$, the confidence threshold is 0.25, and the NMS IOU threshold is 0.5.

**Figure 5:**
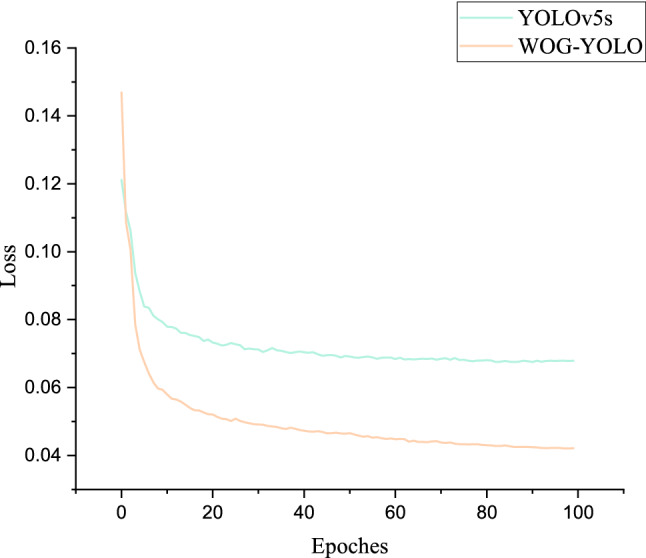
Loss of YOLOv5s and WOG-YOLO in the training processes.

The default and the optimized hyparameters are shown in Table 3. The most representative indicator mAP is used in the evaluation of YOLOv5s, WOG-YOLO, YOLOv7^45^, YOLOX^46^ and Faster-RCNN^2^ and the results are shown in Table 4, the ablation study of WOG-YOLO is shown in Table 5 and the loss curve is shown in Fig. 5. The mAP of YOLOv5s is 92.5$\%$ and the F1-score of YOLOv5s is 90.0$\%$. By adding an extra detection head, its mAP improved by 0.6$\%$. Based on YOLOv5-4heads, its C3 module is replaced by the lightweight G-C3 module (G-YOLO), which reduces the mAP by 0.3$\%$. Compared with the YOLOv5s model, the final WOG-YOLO’s overall mAP increases by 1.7$\%$, its mAP of the pedestrian increases by 2.6$\%$, and its mAP of the cyclist increases by 2.3$\%$. As pedestrians and cyclists have comparatively smaller features than cars, the WOG-YOLO model is more sensitive to small objects and has greater precision.

**Table 3:** The default and optimized hyperparameters in YOLOv5.

Hyperparameter	Default	Optimized
lr0	0.01	0.01243
lrf	0.01	0.01289
Momentum	0.937	0.98000
Weight_decay	0.0005	0.00076
Warmup_epochs	3	3.22050
Warmup_momentum	0.8	0.89787
Warmup_bias_lr	0.1	0.08197
Box	0.05	0.04843
Cls	0.5	0.57263
Cls_pw	1	1.18040
Obj	1	1.20690
Obj_pw	1	0.83334

**Table 4:** Indicators of YOLOv5s, WOG-YOLO and other algorithms.

Model	Indicator	Pedestrain	Cyclist	Car	Total
Faster-RCNN	mAP(0.5)/%	86.2	87.4	92.9	88.8
YOLOX		87.5	91.4	95.1	91.3
YOLOv7		88.2	92.0	96.1	92.1
YOLOv5s		89.2	91.9	96.4	92.5
WOG-YOLO		91.8	94.2	96.6	94.2

**Table 5:** Results of WOG-YOLO with different improvements.

Model	Indicator	Pedestrain	Cyclist	Car	Total
YOLOv5s	Precision/%	92.4	92.5	95.0	93.3
	Recall/%	80.2	87.9	92.5	86.9
	F1/%	85.9	90.2	93.7	90.0
	mAP(0.5)/%	89.2	91.9	96.4	92.5
YOLOv5-4heads	Precision/%	91.7	90.1	94.2	92.0
	Recall/%	81.0	88.6	90.5	86.7
	F1/%	86.0	89.3	92.3	89.0
	mAP(0.5)/%	90.4	93.1	95.9	93.1
G-YOLO	Precision/%	91.7	88.8	94.3	91.6
	Recall/%	81.7	89.9	91.3	87.6
	F1/%	86.4	89.3	92.8	90.0
	mAP(0.5)/%	90.6	91.9	95.9	92.8
WOG-YOLO	Precision/%	94.0	91.3	95.8	93.7
	Recall/%	83.2	88.8	91.4	87.8
	F1/%	88.3	90.0	93.6	91.0
	mAP(0.5)/%	91.8	94.2	96.6	94.2

**Figure 6:**
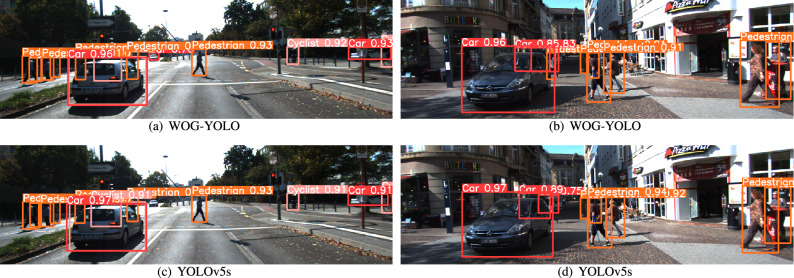
The detection effect of WOG-YOLO and YOLOv5s.

As shown in Fig. 6, WOG-YOLO has excellent capability to detect small objects. Moreover, when part of the object is covered by other things, WOG-YOLO still has reasonable detection ability.

## Conclusion

To accurately identify objects in autonomous driving, a stable and effective detecting algorithm is needed. A novel and efficient optimization algorithm with WOA and GWO is proposed for improving the G-YOLO model.

The hunting strategy of GWO is improved and it’s integrated into WOA, thus the basic structure of MWOA is formed, furthermore, adaptive scaling factor, population concentration ratio, and improved position update method are implemented in MWOA. In comparison with PSO, GWO, WOA, and WhaleFOA, MWOA is verified by different kinds of benchmark functions to have greater precision and better global solving ability.

By replacing the C3 block with the G-C3 block and adding an extra detect layer, the highly optimizable G-YOLO is proposed. To improve G-YOLO’s performance, 12 hyperparameters are optimized by MWOA. The G-YOLO model is trained and evaluated using the self-built dataset containing 5325 images, thus the final whale optimization G-YOLO(WOG-YOLO) model is obtained. Compared with the 92.5$\%$ mAP and 90.0$\%$ F1 in YOLOv5s, WOG-YOLO is 1.7$\%$ better in mAP and 1.0$\%$ in F1. For small objects like pedestrians and cyclists, WOG-YOLO increases the respective mAP by 2.6$\%$ and 2.3$\%$.

In conclusion, the proposed method is an applicable and highly optimized approach to obtain a robust and efficient detection model in autonomous driving.

## Data Availability

The datasets generated and analysed during the current study are available in Kitti.
